# Metabolic Diseases and Down Syndrome: How Are They Linked Together?

**DOI:** 10.3390/biomedicines9020221

**Published:** 2021-02-22

**Authors:** Manon Moreau, Soukaina Benhaddou, Rodolphe Dard, Stefania Tolu, Rim Hamzé, François Vialard, Jamileh Movassat, Nathalie Janel

**Affiliations:** 1 Laboratoire Processus Dégénératifs, Université de Paris, BFA, UMR 8251, CNRS, Stress et Vieillissemen, F-75013 Paris, France; manon.moreau@live.fr (M.M.); soukaina.bnh@gmail.com (S.B.); rodolphe.dard@ght-yvelinesnord.fr (R.D.); 2 Genetics Deptartment, CHI Poissy St Germain-en-Laye, F-78300 Poissy, France; francois.vialard@uvsq.fr; 3 Université Paris-Saclay, UVSQ, INRAE, ENVA, BREED, F-78350 Jouy-en-Josas, France; 4 Laboratoire de Biologie et Pathologie du Pancréas Endocrine, Université de Paris, BFA, UMR 8251, CNRS, F-75013 Paris, France; Stefania.tolu@u-paris.fr (S.T.); Rim.hamze@etu.u-paris.fr (R.H.); jamileh.movassat@u-paris.fr (J.M.)

**Keywords:** down syndrome, obesity, diabetes, immune system, endocrine disorders, thyroid dysfunction, inflammation, central regulation food intake

## Abstract

Down syndrome is a genetic disorder caused by the presence of a third copy of chromosome 21, associated with intellectual disabilities. Down syndrome is associated with anomalies of both the nervous and endocrine systems. Over the past decades, dramatic advances in Down syndrome research and treatment have helped to extend the life expectancy of these patients. Improved life expectancy is obviously a positive outcome, but it is accompanied with the need to address previously overlooked complications and comorbidities of Down syndrome, including obesity and diabetes, in order to improve the quality of life of Down syndrome patients. In this focused review, we describe the associations between Down syndrome and comorbidities, obesity and diabetes, and we discuss the understanding of proposed mechanisms for the association of Down syndrome with metabolic disorders. Drawing molecular mechanisms through which Type 1 diabetes and Type 2 diabetes could be linked to Down syndrome could allow identification of novel drug targets and provide therapeutic solutions to limit the development of metabolic and cognitive disorders.

## 1. Introduction

### 1.1. Down Syndrome

#### 1.1.1. Genotype and Phenotypes

Down syndrome (DS), also known as trisomy 21, is the most common genetic disorder, with a frequency of 1/700 births. It is caused by the presence of an extra copy of human chromosome 21 (HSA21). HSA21 is the smallest chromosome (1% of the genome) with a very low gene density, which explains DS patients can live until adulthood, at the opposite to people with other homogeneous (all cells with the same karyotype) trisomies observed at birth (i.e., trisomy 13 or trisomy 18) [1] who die, for a large majority before one year of life. Other trisomies, at the exception of gonosomes trisomy lead homogeneously to miscarriage.

Three types of trisomy 21 are cytogenetically distinguishable. Free trisomy is defined by 47 chromosomes cells with three HSA21: i.e., 47,XY(XX),+21. This is the most common type of DS (92% of cases). It is well established that advanced maternal age is the major cause of DS occurrence. This trisomy is due to a premature separation of sister chromatids during the first meiotic division [2] which frequency increases with age. Thus, higher maternal age is a major risk factor of DS and all other chromosome aneuploidies. Less frequently, DS results from a Robertsonian translocation [3], mainly the rob(14;21)(q10;q10). The karyotype is then: 46,XY(XX),rob(14;21)(q10;q10),+21. The translocation is often inherited but there are 5–10% of de novo cases [4]. These two types of trisomy could be homogenous (observed in all cells) or with mosaicism (coexistence of both normal cells and trisomic cells). The mosaic DS is caused by a chromosome segregation defect or trisomy rescue during early embryonic development (post zygotic events). Lastly, DS with partial trisomy of the long arm of HSA21 is observed (1% of DS), resulting from an unbalanced reciprocal translocation or partial chromosome duplication.

Constant and well-recognizable symptoms of DS are intellectual disability (ID) and morphological anomalies. DS people have ID with specific cognitive patterns (global mental retardation with motor and language retardation, learning disabilities, weak working memory, good visual memory, joyful spirit, and rare behavioral disturbances) caused by central nervous system defects. Previous studies have demonstrated a decrease of neural precursor cells, early cell cycle exit, unbalanced precursor differentiation in favor of glial cells, increased neuronal death, and migration defects. Moreover, remarkable morphological abnormalities are associated with DS (facial gestalt, single palmar crease, sandal gap, etc.). DS is also associated with several other symptoms and disorders such as congenital heart disease, West syndrome, leukemia, Alzheimer’s disease, and metabolic diseases, such as diabetes and obesity [5].

Fortunately, the majority of these features are not present simultaneously in all individuals with DS, leading to diverse phenotypes with different degrees of severity. Indeed, each person presents a combination of phenotypic characteristics of their own. This phenotypic variability is associated with variability in the expression of genes that are located on HSA21 [6]. It has been proposed that the presence of the extra copy of HSA21 leads to gene dosage perturbation: the “gene dosage effect” hypothesis proposes that dosage imbalance of specific genes or sets of genes on HSA21 causes specific DS phenotypes. Theoretically, the presence of the extra HSA21 should lead to overexpression of all genes encoded on this chromosome. However, some studies have demonstrated that the extra copy of genes do not correlate perfectly with transcript levels. Some complex molecular mechanisms could be involved. It was suggested that some genes are overexpressed, but the expression of a majority of genes located on HSA21 are compensated for by diverse mechanisms [7]. The expression levels of the majority of these genes are still unknown and misunderstood. Furthermore, cases of partial HSA21 trisomy provided strong evidence that not all HSA21 loci are required for the manifestation of DS and have helped to establish genotype–phenotype correlations and led to map genes or regions that constitute a risk. It has been proposed that there is a critical region (DSCR: down syndrome critical region) on the long arm of HSA21, which can be associated to one third of features of DS [8]. This hypothesis predicts that genes in this region are sufficient to produce the main DS phenotypes. However, it has been demonstrated, due to mice trisomic or monosomic mice for the mouse chromosome segment orthologous to the DSCR that while this region is important for features of DS, it is not sufficient [9]. Other regions are involved in DS features, in particular telomeres of HSA21, which can be subjected to triplication in many regions. Thus, DS results from gene dosage imbalance and the interaction between genes located on HSA21 and between these genes and other genes in the genome.

Regardless of the number of gene copies, the presence of the extra HSA21 could also disrupt the whole genome. It leads to alteration in DNA position, changing some DNA–proteins interactions, and therefore impairing the whole genome. As mentioned before, transcriptomic analysis has shown that not all genes in HSA21 are overexpressed. Moreover, there is a global gene expression disturbance in DS [10,11], resulting in dysregulation of the expression of other genes in the genome. This global alteration of gene expression is at the origin of some of the DS phenotypes, and also could explain the phenotypic variability observed between DS individuals [8]. Thus, to understand DS, it is crucial both to understand the genomic content of HSA21 and to evaluate how the expression levels of other genes are altered by the presence of a third copy of HSA21 [12].

#### 1.1.2. DS Comorbidities

Obesity

Obesity is a common condition of clinical and public health importance in many countries around the world. Obesity is the result of a disruption in energy balance leading to an abnormal or excessive fat accumulation in adipose tissue, which leads to health disadvantages and reduced life expectancy [13]. The amount of excess fat and its distribution in the body have important health implications. Indeed, the gynoid obesity, characterized by excess fat peripherally around the body, is less dangerous than android (or abdominal or central) obesity. Abdominal obesity leads to a rise of circulating fatty acids, associated inflammation, and ectopic storage of triglyceride (into liver, muscles, etc.) [14].

Causes of obesity are numerous and complex. Obesity is considered as a multifactorial disease influenced by the interplay of genetic and environmental factors, leading to epigenetics and metagenomics disturbances. Nevertheless, it is generally admitted that the more important cause of obesity is environmental, including the consumption of unhealthy diets (high fat and/or high sugar diet) and the lack of physical exercise. Other environmental factors seem also to have a high impact such as the social class, with a higher prevalence of obesity in low-income households [15]. Psychological factors are also important. Indeed, some psychological problems are counterbalanced by excessive consumption of food. Additional important environmental factors include air pollution or endocrine disruptors [16].

If the development of obesity has an evident environmental contribution, a genetic susceptibility component is also needed [17] and explains the ethnic variability. According to the thrifty gene’s hypothesis [18], some genes contribute to efficiently collect and process food to deposit fat during periods of food abundance (after hunting) in order to provide for periods of food shortage (feast and famine). Currently, while the access to food is almost permanent, these genes are still included in the human genome and involved in the storage of energy, which could participate in the imbalance of energy homeostasis and contribute to obesity. However, this hypothesis has received various criticisms and several modified or alternative hypotheses have been proposed [19,20]. A number of monogenic mutations has also been shown to be involved in the development of obesity. Leptin is a hormone produced by the adipose tissue proportionally to the number of adipose cells, which have an important hypothalamic effect on satiety and energy expenditure. Pathological single nucleotide variation (SNV) in the leptin gene, in the gene encoding the leptin receptor or a mutation located in the leptin-melanocortin pathway have been identified and shown to be at the origin of severe early-onset obesity [21,22,23].

Severe obesity can also be associated with genetic syndromes with neurodevelopmental abnormalities, and other organ/system malformations (for example the Prader–Wili syndrome). Finally, there are some cases of polygenic obesity caused by cumulative contribution of many genes whose effects are amplified in a “weight gain promoting” environment [24]. To date, about 50 genes have been listed as associated with obesity, most with small effects, but in combination with environmental factors, those effects can be amplified. Moreover, some studies show that epigenetic effects can be involved in obesity. Environmental exposures during critical periods of human development can cause permanent changes in a gene’s activity through epigenetic modifications, without changing the sequence of the gene itself [25].

Diabetes Mellitus

Diabetes is a group of metabolic disorders characterized by a chronic high blood sugar level, induced by defects in insulin production, secretion, and/or action. Insulin is an anabolic hormone produced by β cells of the pancreatic islets. This hormone has pleiotropic actions, but one of the most important is the promotion of glucose uptake from the blood, by target tissues (muscles, liver, and adipose tissue), thus leading to the normalization of blood glucose levels after a meal [26,27,28]. Diabetes mellitus is a chronic devastating disease and one of the major public health issues. Diabetes is a heterogeneous disease: the severity and the onset age differ between individuals. Chronic hyperglycemia triggers long-term damage and dysfunction, leading to the failure of different organs, such as eyes, kidneys, nerves, heart, and blood vessels [29]. Clinically, there are two main types of diabetes.

1-Type 1 diabetes (T1DM) is an autoimmune disease due to the destruction of pancreatic β cells and it represents 5–10% of diabetes cases. T1DM usually appears during childhood or adolescence. The presence of various autoantibodies against pancreatic islet cells is the hallmark of the disease. To date, insulin therapy is the only efficient treatment for T1DM [30]. Combinations of many genes contribute to susceptibility to the T1DM. By far, the most important genes are HLA genes (or haplotypes). It has been proposed that the major genetic susceptibility determinants to autoimmune disease are the highly polymorphic HLA (haplotype leukocyte antigen) class II [31].

2-Type 2 diabetes (T2DM) is the most common form of diabetes and represents more than 90% of cases. The prevalence of T2DM rises at an alarming pace, mainly due to poor lifestyle (high fat/high sugar diets and sedentary lifestyle), combined with deleterious environmental contributors (exposure to road traffic (noise and fine particulate matter), low mood/stress/depression, and infection with hepatitis C virus or *Chlamydia pneumoniae*). T2DM is characterized by insulin resistance associated with progressive decrease of insulin secretion and reduction of β cell number [32,33,34]. In addition to these defects, systemic and tissue specific inflammation develops during the course of the disease and aggravates both insulin resistance and β cell dysfunction [35,36,37].

DS, Obesity, and Diabetes

Obesity is more prevalent in individuals with DS than in the general population [38,39,40]. This is also the case in individuals with intellectual disabilities not associated with DS. It is very difficult to identify the exact causes leading to increased risk of obesity in DS. However, it could be in part due to reduced physical activity and unhealthy dietary habits, in addition to endocrine disorders described in DS patients [41].

Even though a higher prevalence of diabetes is observed in the DS population, there are only a few studies addressing the causal association between diabetes and DS. An important issue is to identify the type of diabetes in DS individuals. Some studies argue for T1DM [38], but others support DS association with T2DM/obesity at early age [42]. Taken together, while it has been established that there is a link between DS and metabolic diseases, particularly obesity and diabetes, our comprehension of this association remains limited.

### 1.2. Statement of Hypothesis

**Figure 1:**
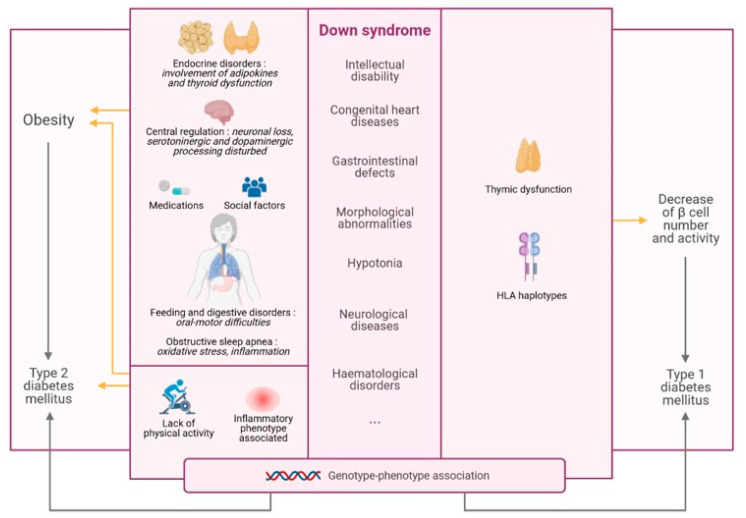
Link between Down syndrome (DS) and metabolic diseases.

Our aim is to discuss the several hypotheses proposed for the association of DS with obesity and diabetes and to apprehend the molecular mechanisms through which T1DM and T2DM could be linked to DS (Figure 1).

## 2. DS and Obesity

### 2.1. Increased Prevalence of Obesity in DS

Most studies on this issue are based on the body mass index (BMI) and conclude that the rate of overweight and obesity is two to four times higher in adults and children with DS compared to general population. Of DS individuals 33% to 71% are concerned with obesity [43,44]. Gender and age seem to have an influence in obesity in individuals with DS. BMI is higher in girls compared to boys in DS children [45] and higher in youth than adults. The shift in the BMI curve occurs at the age of 3 years for girls and 5 years for boys, with a rapid BMI increase at around 14 years old [46]. Females with DS show higher levels of fat, and lower levels of lean mass, assessed by dual energy X-ray absorptiometry (DEXA), compared to males with DS [47]. The percentage of body fat (PBF) measured by bioelectrical impedance is higher in young people with DS [48]. According to Osaili et al., PBF is elevated in female DS children and adolescents compared to males [49]. In adults, a study shows no difference in percentage of obesity between male and female DS individuals [43]. However, in a recent study, higher PBF was observed in female DS children compared to DS males, but a higher prevalence of overweight and obesity, based on BMI, is observed in DS males [50] pointing to a possible bias in the determination of overweight and obesity in DS by using BMI or PBF as methodology.

Regarding the lipid profile, an increase in cholesterol, low density lipoprotein (LDL), and high density lipoprotein (HDL) is observed in DS children [51] and in adults [52], compared to individuals without DS. Another study reports a high frequency of dyslipidemia—low HDL-cholesterol, hypertriglyceridemia, and a combination of both (atherogenic dyslipidemia), in DS children [53] thus increasing cardiovascular risks for these individuals.

### 2.2. Physical Activity in DS

One explanation currently used to explain overweight and obesity in DS is their lifestyle, particularly the lack of physical activities. Pitchford et al. show an association between a higher level of adiposity and low levels of physical activity in adolescents with DS [54]. Adults with DS also exercise less. DS individuals present a reduced exercise capacity due to a lower peak of oxygen uptake (VO_2peak_) [55] probably because of ventilatory dysfunction, deficits of the cerebellum, muscle hypotonia, and ligamentous laxity that may impact the control of body dynamics and balance [56,57,58,59]. Exercise programs have been tested in DS individuals. These studies suggest health benefits for patients, with improved cardiometabolic risk profile, muscle strength, aerobic capacity, proprioception, and postural stability [60]. However, another study reported no efficacy of exercise to achieve weight loss in people with DS [61].

### 2.3. Endocrine Disorders in DS

#### 2.3.1. Involvement of White Adipose Tissue—Adipokines

Obesity is associated with body fat accumulation that leads to disturbances in secretion of adipokines. Leptin regulates appetite and body weight. When body fat increases, it leads to a dysregulation of the appetite, increased leptin levels [62] and the development of leptin resistance [63,64]. In non-obese patients with DS, leptin levels are higher [65,66] compared to controls suggesting an association between DS and leptin resistance. In DS children, circulating leptin is increased compared to adult and old DS individuals [67]. In a cohort of obese Egyptian children with DS, leptin levels were increased compared to non-obese DS children [68]. These results suggest a probable inherent genetic basis for hyperleptinemia and leptin resistance in DS. It has been shown that Ts65Dn mice consumed more calories and presented increased leptin levels, suggesting that leptin would be ineffective in controlling the satiety [69]. The analysis and comparison with other DS mouse models should allow us to dissect the region of chromosome 21 containing genes related to leptin resistance.

Adiponectin is involved in the control of glucose metabolism [70,71] and has anti-inflammatory properties [72,73,74]. Adiponectin levels decrease in case of body fat accumulation [62]. In non-obese DS children, adiponectin levels tend to be lower than controls [75] and lower in DS children and adults compared to older DS individuals [67].

There is a lack of data suggesting other possible disturbances in hormones that regulate appetite and weight gain in DS patients. One study however, using Ts65Dn mice, showed a decrease in ghrelin levels [69]. This hormone, produced by the stomach, is a circulating orexigenic factor [76]. Ghrelin secretion was shown to be impaired in obesity and insulin resistance and has been found to be reduced in obese human subjects [77].

#### 2.3.2. Thyroid Dysfunction in DS

Thyroxine (T4) is the major secretory product of the thyroid gland and it is a precursor of the active form of the thyroid hormone, the 3,5,3′-triiodothyronine (T3) [78]. Thyrotropin releasing hormone (TRH) and thyroid stimulating hormone (TSH), produced by hypothalamus and hypophysis, respectively, regulate thyroid hormone (TH) production and release. A negative feedback loop by TH acts on TSH and TRH secretion [79]. TH closely interacts with energy metabolism. TH status correlates with body weight through the regulation of energy expenditure. It contributes to appetite regulation and maintenance of optimal energy balance by a communication with white adipose tissue and brain via leptin [80,81]. TH is also involved in cholesterol metabolism, glucose homeostasis via modulation of gene expression, and thermogenesis [82,83]. Hypothyroidism leads to reduced gluconeogenesis and lipolysis, increased cholesterol levels, and weight gain [84,85,86].

Individuals with DS have higher prevalence of thyroid dysfunction, which is the most common endocrine abnormality in DS. The rate of thyroid dysfunction is 25–38 fold higher in DS compared to the general population [87,88]. It includes congenital hypothyroidism, subclinical hypothyroidism, autoimmune and non-autoimmune acquired hypothyroidism, and hyperthyroidism [89]. Hypothyroidism, the most frequent thyroid dysfunction in DS, is characterized by a decrease in TH secretion and elevated TSH levels. TH levels could remain normal because they are compensated by the elevation of TSH. Nonetheless, hypothyroidism is well known in DS, supervised during childhood and treated with levothyroxine. Since hypothyroidism is associated with increased weight gain in the general population, its higher prevalence in DS individuals could contribute to higher prevalence of obesity in DS patients.

### 2.4. Feeding and Digestive Disorders

DS can lead to physical defining features that could affect the feeding in individuals with DS. Muscular hypotonia is a constant characteristic of this disease. Hypotonia of the tongue leads to an incorrect positioning of teeth [90]. The tongue is thick and put in place at the bottom of mouth. This positioning can generate an inadequacy in the development of the palate. Breast feeding may be difficult for DS babies and a baby’s bottle should be adopted [91]. During childhood, taste buds hypertrophy and fissures can appear on tongue [92,93]. It can affect the sense of taste and there is an increased susceptibility to inflammation and infection. These problems can alter chewing and swallowing. In addition, dental anomalies are current in DS. Some teeth, temporary or definitive, can be missed or be malformed in DS [94].

Esophageal dysmotility syndromes could also affect feeding behavior. Furthermore, gastroesophageal reflux disease (GERD) is current in DS but not more to the general population. One study shows that children with GERD consume a larger amount of fat, more calories, and less dietary fiber compared to controls with the same age and weight [95]. Achalasia, due to a defect of enteric neurons of esophagus, is also more frequent in DS than general population [96]. It is characterized by dysphagia, a discomfort of swallowing for 99% patients, regurgitation, and vomiting [97].

Together, all these oral-motor difficulties can affect the feeding behavior and can influence the choice of food such as high-fat diet. A study reports narrow food preferences and texture selectivity in DS children [98]. In the study of Nordstrøm et al., authors reported that adolescent and adult DS patients eat less fruits and vegetables compared to patients with other intellectual disabilities [99]. Due to insufficient mastication associated with rapid swallowing without chewing, DS people can develop a secondary bulimia. In a study of 83 children and adolescents with DS, food selectivity represents 62.2% of feeding difficulties, continued eating in the presence of food 57.7% and swallowing without sufficient chewing 50% [49].

### 2.5. Inflammation

The association between obesity and chronic low-grade inflammation has been established from both clinical and experimental studies. Nevertheless, it has not been explored in DS. Using Ts65Dn mice, a study shows hyperglycemic and hypoinsulinemic phenotype associated with adiposity, increased inflammatory biomarkers, and a global state of low-grade inflammation [69]. In a meta-analysis, DS children exhibited increased levels of proinflammatory (interleukin IL-2 and IL-6) and anti-inflammatory cytokines (IL-10 and interleukin 1 receptor antagonist (IL-1RA)) [100]. Tumor necrosis factor alpha (TNF-α), IL-1β, and interferon gamma (IFN-γ) levels were also higher in children and adults with DS [100]. These results suggest an inflammatory phenotype associated with DS that may contribute to obesity.

### 2.6. Obstructive Sleep Apnea

Obstructive sleep apnea (OSA) is a sleep-disordered breathing characterized by periodic reductions in airflow during sleep due to partial or complete obstruction of upper airways [101]. There are many factors that can increase the risk to develop OSA, including obesity, cardiovascular complications, and diabetes [102]. Reciprocally, OSA has a role in the development of obesity [103]. In non-obese apneic patients, some studies report leptin and insulin resistance [104,105]. Due to hypoxia induced by intermittent respiratory arrest, OSA stimulates oxidative stress and inflammation [106,107], which could contribute to maintain obesity. OSA is a common disorder in DS. It affects at least 50% of DS children [108] and nearly 100% of adults [109], compared to 5–8% of general population [110]. This respiratory syndrome could be a contributor to obesity in DS.

### 2.7. Social Factors

Social environment has an influence on food behavior. Poor socioeconomic status is associated with overweight and obesity in general population [111]. However, there is no study on the impact of social and economic environment on obesity in individuals with intellectual disabilities. DS patients in institutions could be less susceptible to develop obesity, compared to patients who live with their family or independently, because of balanced meals.

One study in a population of young adult DS women shows a significant correlation, between their BMI and the BMI of their family, especially of their mother, probably due to familial food habits and obstacles to physical activities [112]. Additionally, parents reported to be less strict regarding food with their DS children compared to their siblings [113]. Families need support to adapt their food habits and DS individuals can learn appropriate eating habits by imitation of parents at the earliest ages.

### 2.8. Medications

Psychiatric disorders are associated with DS. They include major depressive disorder, bipolar and anxiety disorders, obsessive-compulsive disorder, attention-deficit/hyperactivity disorder, autism spectrum disorder, psychosis, and catatonia [114]. These disorders require psychotropic medications like antidepressants, antipsychotics, and antiepileptic drugs, which are known to be associated with weight gain [115].

### 2.9. Central Regulation of Food Intake

DS is characterized by neuronal loss in brain due to higher neuronal apoptosis, reduced neuronal division during development and dysregulation of astrocyte-to-neuron ratio [116]. Hypothalamus is one of the cerebral structures that regulate appetite and energy balance. It consists of different neuronal populations assembled in structures called nuclei. They are involved in reproduction, thermoregulation, growth, circadian rhythm, and food intake [117]. In the arcuate nucleus (ARC), there are two populations of neurons, which regulate food intake and energy expenditure. One population coexpresses neuropeptide Y (NPY) and agouti-related protein (AgRP) and stimulates food intake [118]. The other population coexpresses proopiomelanocortin (POMC) and cocaine- and amphetamine-related transcript (CART) and inhibits appetite [119,120]. These neurons receive afferent signals from gut, brain, and white adipose tissue and transmit information via neuronal projections to hypothalamic areas such as dorsomedial nucleus (DMN), paraventricular nucleus (PVN), ventromedial hypothalamic nucleus (VMN), and lateral hypothalamic area (LHA), involved in the control of appetite [121,122,123]. One study reports a decrease in the number of neurons in the ARC of hypothalamus in DS patients [124], suggesting that neuronal loss could lead to the disruption of hypothalamus regulation of appetite in DS and may contribute to obesity.

Different brain structures are involved in the reward system. The central component of this system is the mesocorticolimbic pathway. It consists of dopaminergic neurons on the ventral tegmental area (VTA), which send projections to the nucleus accumbens (NAc) and prefrontal cortex. Other regions of brain are involved in reward response like hippocampus and amygdala. The neurotransmitter dopamine mediates emotions and pleasure and is also known to mediate behaviors and to participate in the control of impulsive choices and perseveration [125]. Food activates these structures by an increase in dopamine release and might be considered like an addiction, to modify consummatory behaviors [126,127]. There are few studies on DS patients showing reduced dopamine levels in the postmortem brain of older DS patients [128] and reduced monoamines in DS adults [129]. Moreover, some studies showed a relationship between the dual specificity tyrosine-phosphorylation-regulated kinase 1 (DYRK1A), a serine/threonine kinase overexpressed in DS, and the reward system. DYRK1A is strongly expressed in striatum, which receive dopamine projections [130] implicated in compulsive behaviors [131]. In a model of transgenic mice overexpressing DYRK1A, the authors show alteration in serotoninergic and dopaminergic processing in the brain [132], but the involvement of DYRK1A in reward-related mechanisms in the control of food intake should be further investigated. Collectively, these data suggest that the control of food intake by reward system could be altered in DS.

## 3. DS and Diabetes Mellitus

From the last 30 years, some epidemiological studies have reported increased prevalence of diabetes in DS population compared to the general population. However, these studies present some limitations. In particular, they did not distinguish the various forms of diabetes mellitus in DS people [133], the age distribution, nor reported the glycemia, only considering the glucosuria [134]. Finally, no clinical or biochemical information regarding diabetic characteristics were reported. It remains to determine which type of diabetes mellitus appears in DS, and what are the molecular mechanisms involved.

### 3.1. T1DM and DS

It has been reported that T1DM prevalence is 4 times higher in DS people compared to the general population in Denmark [135]. Moreover, it has been suggested that T1DM appears earlier in DS patients than in the general population, with a peak of T1DM of around 8 years, compared to 14 years in the general population [136].

#### 3.1.1. T1DM in DS: A Link with Immune System Defects

It is well-known that defects of the immune system are increased in DS, and it might explain the rise of prevalence of autoimmune diseases in these people. Indeed, immune disorders such as autoimmune diseases (thyroid disease, coeliac disease, or T1DM), myeloid acute leukemia, or repeated infections of higher airways in DS population are higher compared to the general population, and various other immunological abnormalities are found in DS people [38,89,137,138,139,140].

Thymus has an important role in immunity, including the negative selection, which consists of destruction of self-reactive T cells [141]. Medullary thymic epithelial cells (mTEC) express and present tissue-specific peripheral antigens, under the control of transcriptional regulators such as autoimmune regulator (AIRE) protein, to select autoreactive T cells [142]. In DS, a smaller size and abnormal structure of thymus are reported in patients [143]. The AIRE gene is located on chromosome 21 and it has been established that the expression of AIRE is reduced in DS patients [144,145]. This leads to a decrease in the expression of tissue-specific antigens and could contribute to a defect of negative selection of T cells and thus promote autoimmunity in DS. Such an autoimmune context could be involved in the development of T1DM. While many differences between the immune system of DS people and non-DS people have been reported, the defects of immune system, which leads to the higher prevalence of T1DM in DS, are still misunderstood.

#### 3.1.2. Genotype–Phenotype Association and T1DM-DS

HLA genes are located on chromosome 6 and are involved in the presentation of antigen to T-cells. HLA genes encode for proteins that are key mediators of immune responses to pathogens, and the development of self-tolerance. In the case of T1DM, HLA encoded by the locus DQ (combinate with DR region) are a hallmark of T1DM predisposition [146]. Aitken et al. examined the frequency of diabetes-associated high-risk HLA haplotypes in children with DS or T1DM diagnosed before the age of 21 [147]. They reported an increase of diabetes-associated HLA class II genotypes in children with DS and T1DM compared to controls. This suggests that HLA susceptibility in autoimmune diabetes might be the same in DS patients and in T1DM patients. Notwithstanding, the same study showed that DS children with T1DM were less likely to carry the highest-risk genotype DR4-DQ8/DR3-DQ2 than children with T1DM from the general population and more likely to carry low-risk (DR2-DQ6/X or X/X) genotypes. The authors suggest that the explanation for less HLA risk in DS children with T1DM might be the existence of an HLA-dependent and an HLA-independent etiology of diabetes in DS. Understanding how autoimmunity occurs in the absence of HLA risk genotypes is very important for the comprehension of increased prevalence of T1DM in the DS population. Further investigations are needed to identify genetic variants on HSA21 that may increase the prevalence of T1DM in DS patients.

Some of the genes located on HSA21 might be directly associated to the development of autoimmune diseases and/or more specifically to T1DM. Amyloid precursor protein (APP), involved in the development of Alzheimer’s disease in DS people, is at the origin of the amyloid deposit in the pancreas of diabetic patients [148,149], leading to tissue destruction and tissue inflammation [150]. Superoxide dismutase 1 (SOD1) is probably involved in inflammation and immune abnormalities [151]. Therefore, it is possible that polymorphisms in some of these genes could predispose to autoimmune disease in combination with other gene variants. Additionally, it has been suggested that in DS, deregulation of genes encoded by HSA21 might impair interactions between immature thymocyte and thymic stromal cells, which might explain the immune defects in DS [152]. It has also been proposed that immunodeficiency might be due to metabolic or nutritional factors, particularly zinc deficiency [153]. Moreover, the defects of the immune system can lead to an increased susceptibility to infections and it may make people with DS more susceptible to autoimmune diseases. Increased risk of viral infections can also induce T-cells to mistake viral antigen for β cells antigen. Nevertheless, further studies are necessary to resolve the underlying mechanisms of the impaired immune system in DS people.

### 3.2. T2DM and DS

The association between DS and T1DM, as an autoimmune disease is clearly established. However, the association between DS and T2DM is poorly documented.

#### 3.2.1. T2DM in DS: A Link with Obesity

Insulin resistance, metabolic syndrome, and early T2DM are relatively common in DS subjects due to premature ageing, obesity (associated to increased inflammation), and sedentary lifestyle. Moreover, it seems that fat distribution in DS people is different, more truncal than peripherical, which results in muscle hypotonia in DS people [154]. As fat is more stored in the abdominal region, it may contribute to insulin resistance and consequently to the development of T2DM. Therefore, the distribution of fat stores seems to be more important in the development of T2DM in DS people than the obesity itself. In the case of obesity, the development of adipose tissue is associated with the development of vascularization. However, adipose tissues proliferate more quickly than vascularization, which leads to hypoxia and cell apoptosis, and extended inflammation. The inflammation of visceral adipose tissue can lead to insulin resistance, which precedes T2DM. It has been demonstrated that chronic inflammation is associated with T2DM, and certain proinflammatory cytokines like TNF-α are involved in the development of insulin resistance, leading to diabetes. It might be suggested that diabetes in DS people is due to the higher level of proinflammatory cytokines such as TNF-α and IL-6, as it had been reported in a few studies in non-obese children and adult DS individuals respectively [155,156]. However, other studies demonstrated that the level of proinflammatory cytokines is decreased in DS people [157]. More studies are needed to better analyze the levels and the nature of proinflammatory cytokines in DS.

#### 3.2.2. Genotype–Phenotype Association and T2DM-DS

In addition to the extrapancreatic components of T2DM described above, it is now generally admitted that decreased number of β cells and impaired insulin secretion are crucial in T2DM pathogenesis [158]. Therefore, in recent years, several studies have been conducted to determine whether genes in HSA21 are associated with β cell deficits. S100B is one of these genes. The calcium binding protein S100B is one of the S100 family proteins involved in many cellular processes such as cytoskeletal dynamic, intracellular calcium homeostasis, proliferation, differentiation, immune homeostasis, and inflammation [159,160,161,162]. The gene that encodes S100B is overexpressed in DS. A study in S100B knock-out (KO) mice showed that these mice are resistant against experimental diabetes induced by streptozotocin, compared to wild-type mice. S100B KO mice show less hyperglycemia, higher glucose tolerance and insulin sensitivity, and reduced β cell death, but with similar levels of insulin compared to controls [163]. These results suggest that S100B could play a role in the development of diabetes.

Pieris et al. have recently reported that the regulatory of calcineurin 1 (RCAN1) gene, one of the genes located in DSCR, is associated with β cell mitochondrial dysfunction, resulting in reduced ATP production and hence altered insulin secretion [164]. It has been suggested that mitochondrial dysfunction could contribute to increased susceptibility of individuals with DS to diabetes [165]. Another gene potentially linking DS to impaired β cell mass and therefore to T2DM is the gene encoding DYRK1A. During the last few years, several studies have demonstrated that inhibition of DYRK1A kinase activity stimulates human β cell proliferation [166,167,168], suggesting that this enzyme might act as a negative regulator of β cell growth. Therefore, DYRK1A could be a potential candidate linking DS to impaired functional β cell mass in T2DM. The development of an individual’s complement of β cells begins during embryonic life and undergoes a rapid postnatal expansion, largely accomplished through replication of the existing β cells [169]. It has been suggested that a risk factor for the development of diabetes might be a failure to establish a sufficient β cell mass during infancy [170]. β cell replication requires nuclear translocation of the transcription factor nuclear factor of activated T cells (NFAT). Nuclear translocation of NFAT is activated by the phosphatase calcineurin and inhibited by NFAT kinases, which includes DYRK1A and glycogen synthase kinase 3 beta (GSK3β) [166]. In this sense, aminopyrazine treatment induces robust β cell proliferation, most likely as a result of combined inhibition of DYRK1A and GSK3β [164], GSK3β being a target of DYRK1A kinase activity [171]. A series of molecules developed as potent GSK3 β inhibitors, also inhibiting DYRK1A, induced nuclear retention of NFAT in β cells [172]. However, a study by Butler et al. aimed to establish a potential congenital deficit in β cell proportion in post-mortem pancreatic tissues of people with DS, reported no difference in β cell population in the pancreases of the DS group compared to that of control subjects [173]. It should be noted that this study was conducted in a rather small number of subjects and there were no diabetic subjects among the donors. Therefore, further investigations are needed to determine potential defects in the endocrine pancreas of people with DS.

The elucidation of this question remains difficult, mostly because of the lack of non-invasive approaches for the assessment of the β cell mass in living humans. Preclinical models of DS in rodents have been helpful to establish the links between DS and metabolic disorders. Recent studies conducted in TS65dn mice showed that these animals have increased fat mass and energy intake along with increased leptin levels compared to wild-type mice [69]. Moreover, circulating markers of inflammation were increased in these mice [69]. Peiris et al. have also reported higher basal glycemia in two mice models of DS: Ts65Dn mice and Dp16 mice [174]. Dp16 mice contain also a partial duplication of the mouse chromosome 16, but unlike Ts65Dn mice, they contain a duplication of only the parts of chromosome 16 that are homologous to HSA21. Their study thus provided a region of HSA21 containing genes that cause hyperglycemia and suggested a link between DS and abnormal glucose and energy metabolism. Further investigations are needed to determine whether some of the genes with abnormal expression in DS could be linked to defective β cell proliferation and/or secretory function, thus increasing the risk of diabetes.

## 4. Conclusions

It is well established that the prevalence of T1DM is higher in DS population compared to the general population. This seems to be due to the disorders of immune system in DS, caused by genetic and environmental factors, although mechanisms involved still need to be better defined. Moreover, genes located on HSA21 that confer a specific susceptibility to T1DM in DS patients, need to be further investigated.

On the other hand, while higher rates of overweight and obesity are consistently reported in individuals with DS [175], the association between DS and T2DM has not been thoroughly investigated, and references on insulin resistance indexes in DS are scarce in the literature. Recent studies in animal models and human cells, linking genes in HSA21 (RCAN1, DYRK1A, etc.) to β cell growth and secretory function, have opened new research avenues to explore the potential impairment of pancreatic and extra pancreatic metabolic tissues in DS. Numerous studies have demonstrated the beneficial effects of DYRK1A inhibition not only for β cell proliferation but also for cognitive improvement [176]. Finding new molecular targets in order to limit not only the development of diabetes but also ID in DS patients could provide therapeutic solutions for two interconnected conditions: diabetes and cognitive deficits.

In summary, fortunately, with improved medical care for DS individuals, their life expectancy has greatly increased over the last decades. However, given the increased risk of comorbidities including obesity and diabetes in DS, the understanding of mechanisms linking these pathologies, and the identification of effective interventions for this special population is crucial and warrant additional research.

## Data Availability

Not applicable.

## Abbreviations

AgRP	agouti-related protein
APP	Amyloid Precursor Protein
ARC	arcuate nucleus
AIRE	autoimmune regulator
BMI	body mass index
CART	cocaine- and amphetamine-related transcript
DMN	dorsomedial nucleus
DS	Down syndrome
DSCR	Down Syndrome Critical Region
DEXA	dual energy X-ray absorptiometry
DYRK1A	Dual specificity tyrosine-phosphorylation-regulated kinase 1
GERD	gastroesophageal reflux disease
GSK3 β	Glycogen synthase kinase 3 beta
HDL	high density lipoprotein
HLA	Haplotype Leukocyte Antigen
HSA21	human chromosome 21
ID	intellectual disability
IL-	interleukin-
IL-1RA	interleukin 1 receptor antagonist
IFN-γ	interferon gamma
KO	knock-out
LDL	low density lipoprotein
LHA	lateral hypothalamic area
mTEC	medullary thymic epithelial cells
NAc	nucleus accumbens
NFAT	nuclear factor of activated T cells
NPY	neuropeptide Y
OSA	Obstructive sleep apnea
PBF	percentage of body fat
POMC	proopiomelanocortin
PVN	paraventricular nucleus
RCAN1	Regulatory of Calcineurin 1
SNV	single nucleotide variation
SOD1	Superoxide dismutase 1
T1DM	Type 1 diabetes mellitus
T2DM	Type 2 diabetes mellitus
T3	3,5,3′-triiodothyronine
T4	Thyroxine
TH	thyroid hormone
TNF-α	Tumor necrosis factor alpha
TRH	Thyrotropin releasing hormone
TSH	Thyroid stimulating hormone
VMN	ventromedial hypothalamic nucleus
VTA	ventral tegmental area

## References

[1] Lakovschek I.C., Streubel B., Ulm B. (2011). Natural outcome of trisomy 13, trisomy 18, and triploidy after prenatal diagnosis. Am. J. Med. Genet. Part A.

[2] Epstein C.J. (1986). Developmental genetics. Cell. Mol. Life Sci..

[3] Al-Alaiyan S., Al-Omran H., Kattan H., Sakati N., Nyhan W.L. (1995). Down Syndrome and Recurrent Abortions Resulting from Robertsonian Translocation 21q21q. Ann. Saudi Med..

[4] Petersen M.B., Adelsberger P.A., Schinzel A.A., Hinkel G.K., Antonarakis S.E. (1991). Down Syndrome Due to De Novo Robertsonian Translocation t(l 4q;21 q): DNA Polymorphism Analysis Suggests That the Origin of the Extra 21q Is Maternal. Am. J. Hum. Genet..

[5] Antonarakis S.E., Skotko B.G., Rafii M.S., Strydom A., Pape S.E., Bianchi D.W., Sherman S.L., Reeves R.H. (2020). Down syndrome. Nat. Rev. Dis. Prim..

[6] Epstein C.J. (2006). Down’s syndrome: Critical genes in a critical region. Nat. Cell Biol..

[7] Yahya-Graison E.A., Aubert J., Dauphinot L., Rivals I., Prieur M., Golfier G., Rossier J., Personnaz L., Créau N., Bléhaut H. (2007). Classification of Human Chromosome 21 Gene-Expression Variations in Down Syndrome: Impact on Disease Phenotypes. Am. J. Hum. Genet..

[8] Delabar J.-M., Theophile D., Rahmani Z., Chettouh Z., Blouin J.-L., Prieur M., Noel B., Sinet P.-M. (1993). Molecular Mapping of Twenty-Four Features of Down Syndrome on Chromosome 21. Eur. J. Hum. Genet..

[9] Olson L.E., Richtsmeier J.T., Leszl J., Reeves R.H. (2004). A Chromosome 21 Critical Region Does Not Cause Specific Down Syndrome Phenotypes. Science.

[10] Antonarakis S.E., Lyle R., Dermitzakis E.T., Reymond A., Deutsch S. (2004). Chromosome 21 and Down syndrome: From genomics to pathophysiology. Nat. Rev. Genet..

[11] Hervé B., Coussement A., Gilbert T., Dumont F., Jacques S., Cuisset L., Chicard M., Hizem S., Bourdoncle P., Letourneur F. (2016). Aneuploidy: The impact of chromosome imbalance on nuclear organization and overall genome expression. Clin. Genet..

[12] Wiseman F.K., Alford K.A., Tybulewicz V.L., Fisher E.M. (2009). Down syndrome—Recent progress and future prospects. Hum. Mol. Genet..

[13] Ofei F. (2005). Obesity—A Preventable Disease. Ghana Med. J..

[14] Kissebah A.H., Krakower G.R. (1994). Regional adiposity and morbidity. Physiol. Rev..

[15] Klinerogerseva R., Eagle T.F., Sheetz A., Woodward A., Leibowitz R., Song M., Sylvester R., Corriveau N., Kline-Rogers E., Jiang Q. (2015). The Relationship between Childhood Obesity, Low Socioeconomic Status, and Race/Ethnicity: Lessons from Massachusetts. Child. Obes..

[16] Brantley P.J., Myers V.H., Roy H.J. (2005). Environmental and lifestyle influences on obesity. J. La. State Med Soc. Off. Organ La. State Med. Soc..

[17] Herrera B.M., Lindgren C.M. (2010). The Genetics of Obesity. Curr. Diabetes Rep..

[18] Neel J.V. (1999). Diabetes Mellitus: A “Thrifty” Genotype Rendered Detrimental by “Progress”? 1962. Bull. World Health Organ..

[19] Southam L., Soranzo N., Montgomery S.B., Frayling T.M., McCarthy M.I., Barroso I., Zeggini E. (2009). Is the thrifty genotype hypothesis supported by evidence based on confirmed type 2 diabetes- and obesity-susceptibility variants?. Diabetol..

[20] Speakman J.R. (2006). Thrifty genes for obesity and the metabolic syndrome—Time to call off the search?. Diabetes Vasc. Dis. Res..

[21] Dubern B., Clement K. (2012). Leptin and leptin receptor-related monogenic obesity. Biochimie.

[22] Ozsu E., Ceylaner S., Onay H. (2017). Early-onset severe obesity due to complete deletion of the leptin gene in a boy. J. Pediatr. Endocrinol. Metab..

[23] Shabana, Hasnain S. (2016). The p. N103K mutation of leptin (LEP) gene and severe early onset obesity in Pakistan. Biol. Res..

[24] Thaker V.V. (2017). Genetic and epigenetic causes of obesity. Adolesc. Med. State Art Rev..

[25] Herrera B.M., Keildson S., Lindgren C.M. (2011). Genetics and epigenetics of obesity. Maturitas.

[26] DeFronzo R.A., Gunnarsson R., Björkman O., Olsson M., Wahren J. (1985). Effects of insulin on peripheral and splanchnic glucose metabolism in noninsulin-dependent (type II) diabetes mellitus. J. Clin. Investig..

[27] Iozzo P., Geisler F., Oikonen V., Mäki M., Takala T., Solin O., Ferrannini E., Knuuti J., Nuutila P. (2003). 18F-FDG PET Study Insulin Stimulates Liver Glucose Uptake in Humans: An 18F-FDG PET Study. J. Nucl. Med..

[28] Virtanen K.A., Lönnroth P., Parkkola R., Peltoniemi P., Asola M., Viljanen T., Tolvanen T., Knuuti J., Rönnemaa T., Huupponen R. (2002). Glucose Uptake and Perfusion in Subcutaneous and Visceral Adipose Tissue during Insulin Stimulation in Nonobese and Obese Humans. J. Clin. Endocrinol. Metab..

[29] Deshpande A.D., Harris-Hayes M., Schootman M. (2008). Epidemiology of Diabetes and Diabetes-Related Complications. Phys. Ther..

[30] Bhatia E., Aggarwal A. (2007). Insulin Therapy for Patients with Type 1 Diabetes. J. Assoc. Physicians India.

[31] Gregersen P.K. (1989). HLA class II polymorphism: Implications for genetic susceptibility to autoimmune disease. Lab. Investig..

[32] Butler A.E., Janson J., Bonner-Weir S., Ritzel R.A., Rizza R.A., Butler P.C. (2003). -Cell Deficit and Increased -Cell Apoptosis in Humans With Type 2 Diabetes. Diabetes.

[33] Basu A., Man C.D., Basu R., Toffolo G., Cobelli C., Rizza R.A. (2009). Effects of Type 2 Diabetes on Insulin Secretion, Insulin Action, Glucose Effectiveness, and Postprandial Glucose Metabolism. Diabetes Care.

[34] Rizza R.A., Mandarino L.J., Genest J., Baker B.A., Gerich J.E. (1985). Production of Insulin Resistance by Hyperin-sulinaemia in Man. Diabetologia.

[35] Shoelson S.E., Lee J., Goldfine A.B. (2006). Inflammation and insulin resistance. J. Clin. Investig..

[36] Hotamisligil G.S., Shargill N.S., Spiegelman B.M. (1993). Adipose expression of tumor necrosis factor-alpha: Direct role in obesity-linked insulin resistance. Science.

[37] Wang C., Guan Y., Yang J. (2010). Cytokines in the Progression of Pancreaticβ-Cell Dysfunction. Int. J. Endocrinol..

[38] Anwar A.J., Walker J.D., Frier B.M. (1998). Type 1 Diabetes Mellitus and Down’s Syndrome: Prevalence, Manage-ment and Diabetic Complications. Diabet. Med..

[39] Farquhar J. (1969). Early-onset diabetes in the general and the down’s syndrome population. Lancet.

[40] Bell A.J., Bhate M.S. (2008). Prevalence of overweight and obesity in Down’s syndrome and other mentally handicapped adults living in the community. J. Intellect. Disabil. Res..

[41] Hawli Y., Nasrallah M., Fuleihan G.E.-H. (2009). Endocrine and musculoskeletal abnormalities in patients with Down syndrome. Nat. Rev. Endocrinol..

[42] Alexander M., Petri H., Ding Y., Wandel C., Khwaja O., Foskett N. (2015). Morbidity and medication in a large population of individuals with Down syndrome compared to the general population. Dev. Med. Child Neurol..

[43] Prasher V.P. (1995). Overweight and obesity amongst Down’s syndrome adults. J. Intellect. Disabil. Res..

[44] Havercamp S.M., Scandlin D., Roth M. (2004). Health Disparities among Adults with Developmental Disabilities, Adults with other Disabilities, and Adults Not Reporting Disability in North Carolina. Public Health Rep..

[45] Pierce M., Ramsey K., Pinter J. (2019). Trends in Obesity and Overweight in Oregon Children with Down Syndrome. Glob. Pediatr. Health.

[46] Mircher C., Briceño L.G., Toulas J., Conte M., Tanguy M.-L., Cieuta-Walti C., Rethore M.-O., Ravel A. (2018). Growth curves for French people with Down syndrome from birth to 20 years of age. Am. J. Med Genet. Part A.

[47] González-Agüero A., Ara I., Moreno L.A., Vicente-Rodríguez G., Casajús J.A. (2011). Fat and lean masses in youths with Down syndrome: Gender differences. Res. Dev. Disabil..

[48] Loveday S.J., Thompson J.M., Mitchell E.A. (2012). Bioelectrical impedance for measuring percentage body fat in young persons with Down syndrome: Validation with dual-energy absorptiometry. Acta Paediatr..

[49] Osaili T.M., Attlee A., Naveed H., Maklai H., Mahmoud M., Hamadeh N., Asif T., Hasan H., Obaid R.S. (2019). Physical Status and Parent-Child Feeding Behaviours in Children and Adolescents with Down Syndrome in The United Arab Emirates. Int. J. Environ. Res. Public Health.

[50] Shea M.O., Shea C.O., Gibson L., Leo J., Carty C. (2018). The prevalence of obesity in children and young people with Down syndrome. J. Appl. Res. Intellect. Disabil..

[51] Buonuomo P.S., Bartuli A., Mastrogiorgio G., Vittucci A., Di Camillo C., Bianchi S., Marafon D.P., Villani A., Valentini D. (2016). Lipid profiles in a large cohort of Italian children with Down syndrome. Eur. J. Med Genet..

[52] De Asua D.R., Parra P., Costa R., Moldenhauer F., Suarez C. (2014). A Cross-Sectional Study of the Phenotypes of Obesity and Insulin Resistance in Adults with Down Syndrome. Diabetes Metab. J..

[53] De La Piedra M.J., Alberti G., Cerda J., Cárdenas A., Paul M.A., Lizama M. (2017). Alta frecuencia de dislipidemias en niños y adolescentes con Síndrome de Down. Revista Chilena de Pediatría.

[54] Pitchford E.A., Adkins C., Hasson R.E., Hornyak J.E., Ulrich D.A. (2018). Association between Physical Activity and Adiposity in Adolescents with Down Syndrome. Med. Sci. Sports Exerc..

[55] Mendonca G.V., Pereira F.D., Fernhall B. (2010). Reduced exercise capacity in persons with Down syndrome: Cause, effect, and management. Ther. Clin. Risk Manag..

[56] Cockerill C.C., Frisch C.D., Rein S.E., Orvidas L.J. (2016). Supraglottoplasty outcomes in children with Down syndrome. Int. J. Pediatr. Otorhinolaryngol..

[57] Rotmensch S., Goldstein I., Liberati M., Shalev J., Ben-Rafael Z., Copel J.A. (1997). Fetal transcerebellar diameter in down syndrome. Obstet. Gynecol..

[58] Guidi S., Ciani E., Bonasoni P., Santini D., Bartesaghi R. (2010). Widespread Proliferation Impairment and Hypocellularity in the Cerebellum of Fetuses with Down Syndrome. Brain Pathol..

[59] Rigoldi C., Galli M., Mainardi L., Crivellini M., Albertini G. (2011). Postural control in children, teenagers and adults with Down syndrome. Res. Dev. Disabil..

[60] Paul Y., Ellapen T.J., Barnard M., Hammill H.V., Swanepoel M. (2019). The health benefits of exercise therapy for patients with Down syndrome: A systematic review. Afr. J. Disabil..

[61] Bertapelli F., Pitetti K., Agiovlasitis S., Guerra-Junior G. (2016). Overweight and obesity in children and adolescents with Down syndrome—prevalence, determinants, consequences, and interventions: A literature review. Res. Dev. Disabil..

[62] Lubkowska A., Radecka A., Bryczkowska I., Rotter I., Laszczyńska M., Dudzińska W. (2015). Serum Adiponectin and Leptin Concentrations in Relation to Body Fat Distribution, Hematological Indices and Lipid Profile in Humans. Int. J. Environ. Res. Public Health.

[63] Frederich R.C., Hamann A., Anderson S., Löllmann B., Lowell B.B., Flier J.S. (1995). Leptin levels reflect body lipid content in mice: Evidence for diet-induced resistance to leptin action. Nat. Med..

[64] Maffei M., Halaas J.L., Ravussin E., Pratley R.E., Lee G., Zhang Y., Fei H., Kim S., Lallone R., Ranganathan S. (1995). Leptin levels in human and rodent: Measurement of plasma leptin and ob RNA in obese and weight-reduced subjects. Nat. Med..

[65] Proto C., Romualdi D., Cento R.M., Romano C., Campagna G., Lanzone A. (2007). Free and total leptin serum levels and soluble leptin receptors levels in two models of genetic obesity: The Prader-Willi and the Down syndromes. Metab..

[66] Magge S.N., O’Neill K.L., Shults J., Stallings V.A., Stettler N. (2008). Leptin Levels among Prepubertal Children with Down Syndrome Compared with Their Siblings. J. Pediatr..

[67] Corsi M.M., Dogliotti G., Pedroni F., Galliera E., Malavazos A.E., Villa R., Chiappelli M., Licastro F. (2009). Adipocytokines in Down’s syndrome, an atheroma-free model: Role of adiponectin. Arch. Gerontol. Geriatr..

[68] Yahia S., El-Farahaty R.M., El-Hawary A.K., El-Hussiny M.A., Abdel-Maseih H., El-Dahtory F., El-Gilany A.-H. (2012). Leptin, insulin and thyroid hormones in a cohort of Egyptian obese Down syndrome children: A comparative study. BMC Endocr. Disord..

[69] Fructuoso M., Rachdi L., Philippe E., Denis R., Magnan C., Le Stunff H., Janel N., Dierssen M. (2018). Increased levels of inflammatory plasma markers and obesity risk in a mouse model of Down syndrome. Free. Radic. Biol. Med..

[70] Kubota N., Terauchi Y., Yamauchi T., Kubota T., Moroi M., Matsui J., Eto K., Yamashita T., Kamon J., Satoh H. (2002). Disruption of Adiponectin Causes Insulin Resistance and Neointimal Formation. J. Biol. Chem..

[71] Maeda N., Shimomura I., Kishida K., Nishizawa H., Matsuda M., Nagaretani H., Furuyama N., Kondo H., Takahashi M., Arita Y. (2002). Diet-induced insulin resistance in mice lacking adiponectin/ACRP30. Nat. Med..

[72] Okamoto Y., Folco E.J., Minami M., Wara A., Feinberg M.W., Sukhova G.K., Colvin R.A., Kihara S., Funahashi T., Luster A.D. (2008). Adiponectin Inhibits the Production of CXC Receptor 3 Chemokine Ligands in Macrophages and Reduces T-Lymphocyte Recruitment in Atherogenesis. Circ. Res..

[73] Ouchi N., Kihara S., Funahashi T., Nakamura T., Nishida M., Kumada M., Okamoto Y., Ohashi K., Nagaretani H., Kishida K. (2003). Reciprocal Association of C-Reactive Protein With Adiponectin in Blood Stream and Adipose Tissue. Circulation.

[74] Wolf A.M., Wolf D., Rumpold H., Enrich B., Tilg H. (2004). Adiponectin induces the anti-inflammatory cytokines IL-10 and IL-1RA in human leukocytes. Biochem. Biophys. Res. Commun..

[75] Tenneti N., Dayal D., Sharda S., Panigrahi I., Didi M., Attri S.V., Sachdeva N., Bhalla A.K. (2017). Concentrations of leptin, adiponectin and other metabolic parameters in non-obese children with Down syndrome. J. Pediatr. Endocrinol. Metab..

[76] Kojima M., Hosoda H., Date Y., Nakazato M., Matsuo H., Kangawa K. (1999). Ghrelin is a growth-hormone-releasing acylated peptide from stomach. Nat. Cell Biol..

[77] Mittelman S.D., Klier K., Braun S., Azen C., Geffner M.E., Buchanan T.A. (2010). Obese Adolescents Show Impaired Meal Responses of the Appetite-Regulating Hormones Ghrelin and PYY. Obesity.

[78] Gereben B., Zavacki A.M., Ribich S., Kim B.W., Huang S.A., Simonides W.S., Zeöld A., Bianco A.C. (2008). Cellular and Molecular Basis of Deiodinase-Regulated Thyroid Hormone Signaling1. Endocr. Rev..

[79] Chin W.W., Carr F.E., Burnside J., Darling D.S. (1993). Thyroid Hormone Regulation of Thyrotropin Gene Expression. Recent Prog Horm. Res..

[80] Guo F., Bakal K., Minokoshi Y., Hollenberg A.N. (2004). Leptin Signaling Targets the Thyrotropin-Releasing Hormone Gene Promoterin Vivo. Endocrinology.

[81] Mantzoros C.S., Ozata M., Negrao A.B., Suchard M.A., Ziotopoulou M., Caglayan S., Elashoff R.M., Cogswell R.J., Negro P., Victoria L. (2001). Synchronicity of Frequently Sampled Thyrotropin (TSH) and Leptin Concentrations in Healthy Adults and Leptin-Deficient Subjects: Evidence for Possible Partial TSH Regulation by Leptin in Humans. J. Clin. Endocrinol. Metab..

[82] Oppenheimer J.H., Schwartz H.L., Lane J.T., Thompson M.P. (1991). Functional relationship of thyroid hormone-induced lipogenesis, lipolysis, and thermogenesis in the rat. J. Clin. Investig..

[83] Ding Y., Tian Y., Guo M., Liu J., Heng D., Zhu B., Yang Y., Zhang C. (2016). Regulation of glucose transport by thyroid hormone in rat ovary. Cell Tissue Res..

[84] Nedvidkova J., Haluzik M., Bartak V., Dostalova I., Vlcek P., Racek P., Taus M., Behanova M., Svacina S., Alesci S. (2004). Changes of Noradrenergic Activity and Lipolysis in the Subcutaneous Abdominal Adipose Tissue of Hypo- and Hyperthyroid Patients: AnIn VivoMicrodialysis Study. Ann. New York Acad. Sci..

[85] Hayek A. (1979). Unimpaired Gluconeogenesis in Congenital Hypothyroidism. Horm. Metab. Res..

[86] Mullur R., Liu Y.-Y., Brent G.A. (2014). Thyroid Hormone Regulation of Metabolism. Physiol. Rev..

[87] Fort P., Lifshitz F., Bellisario R., Davis J., Lanes R., Pugliese M., Richman R., Post E., David R. (1984). Abnormalities of thyroid function in infants with Down syndrome. J. Pediatr..

[88] Graber E., Chacko E., Regelmann M.O., Costin G., Rapaport R. (2012). Down Syndrome and Thyroid Function. Endocrinol. Metab. Clin. North Am..

[89] Pierce M.J., LaFranchi S.H., Pinter J.D. (2017). Characterization of Thyroid Abnormalities in a Large Cohort of Children with Down Syndrome. Horm. Res. Paediatr..

[90] Cuoghi O.A., Topolski F., De Faria L.P., Occhiena C.M., Ferreira N.D.S.P., Ferlin C.R., De Mendonça M.R. (2016). Prevalence of Dental Anomalies in Permanent Dentition of Brazilian Individuals with Down Syndrome. Open Dent. J..

[91] Pisacane A., Toscano E., Pirri I., Continisio P., Andria G., Zoli B., Strisciuglio P., Concolino D., Piccione M., Giudice C.L. (2003). Down syndrome and breastfeeding. Acta Paediatr..

[92] Shukla D., Bablani D., Chowdhry A., Thapar R., Gupta P., Mishra S. (2014). Dentofacial and Cranial Changes in Down Syndrome. Osong Public Health Res. Perspect..

[93] Cohen M.M., Winer R.A. (1965). Dental and Facial Characteristics in Down’s Syndrome (Mongolism). J. Dent. Res..

[94] Anders P.L., Davis E.L. (2010). Oral health of patients with intellectual disabilities: A systematic review. Spéc. Care Dent..

[95] Borodina G., Morozov S. (2020). Children With Gastroesophageal Reflux Disease Consume More Calories and Fat Compared to Controls of Same Weight and Age. J. Pediatr. Gastroenterol. Nutr..

[96] Zarate N., Mearin F., Hidalgo A.M., Malagelada J.-R. (2001). Prospective evaluation of esophageal motor dysfunction in Down’s syndrome. Am. J. Gastroenterol..

[97] Alagiakrishnan K., Bhanji R.A., Kurian M. (2013). Evaluation and management of oropharyngeal dysphagia in different types of dementia: A systematic review. Arch. Gerontol. Geriatr..

[98] Field D., Garland M., Williams K. (2003). Correlates of specific childhood feeding problems. J. Paediatr. Child Health.

[99] Nordstrøm M., Paus B., Andersen L.F., Kolset S.O. (2015). Dietary aspects related to health and obesity in Williams syndrome, Down syndrome, and Prader–Willi syndrome. Food Nutr. Res..

[100] Huggard D., Kelly L., Ryan E., McGrane F., Lagan N., Roche E., Balfe J., Leahy T.R., Franklin O., Doherty D.G. (2020). Increased systemic inflammation in children with Down syndrome. Cytokine.

[101] Patel S.R. (2019). Obstructive Sleep Apnea. Ann. Intern. Med..

[102] Young T., Skatrud J., Peppard P.E. (2004). Risk Factors for Obstructive Sleep Apnea in Adults. JAMA.

[103] Phillips B.G., Hisel T.M., Kato M., Pesek C.A., Dyken M.E., Narkiewicz K., Somers V.K. (1999). Recent weight gain in patients with newly diagnosed obstructive sleep apnea. J. Hypertens..

[104] Bixler E.O., Vgontzas A.N., Lin H.-M., Have T.T., Leiby B.E., Vela-Bueno A., Kales A. (2000). Association of hypertension and sleep-disordered breathing. Arch. Intern. Med..

[105] Patel S.R., Palmer L.J., Larkin E.K., Jenny N.S., White D.P., Redline S. (2004). Relationship between Obstructive Sleep Apnea and Diurnal Leptin Rhythms. Sleep.

[106] Lavie L. (2009). Oxidative Stress—A Unifying Paradigm in Obstructive Sleep Apnea and Comorbidities. Prog. Cardiovasc. Dis..

[107] Unnikrishnan D., Jun J., Polotsky V. (2015). Inflammation in sleep apnea: An update. Rev. Endocr. Metab. Disord..

[108] Maris M., Verhulst S., Wojciechowski M., Van De Heyning P., Boudewyns A. (2016). Prevalence of Obstructive Sleep Apnea in Children with Down Syndrome. Sleep.

[109] Trois M.S., Capone G.T., Lutz J.A., Melendres M.C., Schwartz A.R., Collop N.A., Marcus C.L. (2009). Obstructive Sleep Apnea in Adults with Down Syndrome. J. Clin. Sleep Med..

[110] Marcus C.L., Brooks L.J., Ward S.D., Draper K.A., Gozal D., Halbower A.C., Jones J., Lehmann C., Schechter M.S., Sheldon S.H. (2012). Diagnosis and Management of Childhood Obstructive Sleep Apnea Syndrome. Pediatrics.

[111] Vazquez C.E., Cubbin C. (2020). Socioeconomic Status and Childhood Obesity: A Review of Literature from the Past Decade to Inform Intervention Research. Curr. Obes. Rep..

[112] Fornieles G., Camacho-Molina A., Rosety M.A., Díaz A.J., Rosety I., Rosety-Rodríguez M., Alvero-Cruz J.R., Rosety M., Ordonez F.J. (2013). Maternal fat mass may predict overweight/obesity in non-instituzionalized women with intellectual disability. Nutrición Hospitalaria.

[113] O’Neill K.L., Shults J., Stallings V.A., Stettler N. (2005). Child-feeding practices in children with down syndrome and their siblings. J. Pediatr..

[114] Palumbo M.L., McDougle C.J. (2018). Pharmacotherapy of Down syndrome. Expert Opin. Pharmacother..

[115] Dent R., Blackmore A., Peterson J., Habib R., Kay G.P., Gervais A., Taylor V., Wells G. (2012). Changes in Body Weight and Psychotropic Drugs: A Systematic Synthesis of the Literature. PLoS ONE.

[116] Vacca R.A., Bawari S., Valenti D., Tewari D., Nabavi S.M., Shirooie S., Sah A.N., Volpicella M., Braidy N. (2019). Down syndrome: Neurobiological alterations and therapeutic targets. Neurosci. Biobehav. Rev..

[117] Flament-Durand J. (1980). The hypothalamus: Anatomy and functions. Acta Psychiatr. Belg..

[118] Clark J.T., Kalra P.S., Crowley W.R., Kalra S.P. (1984). Neuropeptide y and human pancreatic polypeptide stimulate feeding behavior in rats. Endocrinology.

[119] Panksepp J., Reilly P., Bishop P., Meeker R.B., Vilberg T.R., Kastin A.J. (1976). Effects of α-MSH on motivation, vigilance and brain respiration. Pharmacol. Biochem. Behav..

[120] Kristensen P., Judge M.E., Thim L., Ribel U., Christjansen K.N., Wulff B.S., Clausen J.T., Jensen P.B., Madsen O.D., Vrang N. (1998). Hypothalamic CART is a new anorectic peptide regulated by leptin. Nat. Cell Biol..

[121] Leibowitz S.F., Hammer N.J., Chang K. (1981). Hypothalamic paraventricular nucleus lesions produce overeating and obesity in the rat. Physiol. Behav..

[122] Mayer J., Thomas D.W. (1967). Regulation of Food Intake and Obesity. Science.

[123] Elias C.F., Saper C.B., Maratos-Flier E., Tritos N.A., Lee C., Kelly J., Tatro J.B., Hoffman G.E., Ollmann M.M., Barsh G.S. (1998). Chemically Defined Projections Linking the Mediobasal Hypothalamus and the Lateral Hy-pothalamic Area. J. Comp. Neurol..

[124] Wisniewski K.E., Bobinski M. (1991). Hypothalamic abnormalities in Down syndrome. Prog. Clin. Boil. Res..

[125] Ebaik J.-H. (2013). Dopamine Signaling in reward-related behaviors. Front. Neural Circuits.

[126] Palmiter R.D. (2007). Is dopamine a physiologically relevant mediator of feeding behavior?. Trends Neurosci..

[127] Schulte E.M., Avena N.M., Gearhardt A.N. (2015). Which Foods May Be Addictive? The Roles of Processing, Fat Content, and Glycemic Load. PLoS ONE.

[128] Yates C., Simpson J., Gordon A., Maloney A., Allison Y., Ritchie I., Urquhart A. (1983). Catecholamines and cholinergic enzymes in pre-senile and senile Alzheimer-type dementia and down’s syndrome. Brain Res..

[129] Reynolds G., Godridge H. (1985). Alzheimer-like brain monoamine deficits in adults with down’s syndrome. Lancet.

[130] Martí E., Altafaj X., Dierssen M., De La Luna S., Fotaki V., Alvarez M., Pérez-Riba M., Ferrer I., Estivill X. (2003). Dyrk1A expression pattern supports specific roles of this kinase in the adult central nervous system. Brain Res..

[131] O’Connor E.C., Kremer Y., Lefort S., Harada M., Pascoli V.J., Rohner C., Lüscher C. (2015). Accumbal D1R Neurons Projecting to Lateral Hypothalamus Authorize Feeding. Neuron.

[132] London J., Rouch C., Bui L.C., Assayag E., Souchet B., Daubigney F., Medjaoui H., Luquet S., Magnan C., Delabar J.M. (2017). Overexpression of the DYRK1A Gene (Dual-Specificity Tyrosine Phosphorylation-Regulated Kinase 1A) Induces Alterations of the Serotoninergic and Dopaminergic Processing in Murine Brain Tissues. Mol. Neurobiol..

[133] Milunsky A., Neurath P.W. (1968). Diabetes Mellitus in Down’s Syndrome. Arch. Environ. Health Int. J..

[134] Jeremiah D.E., Leyshon G.E., Rose T., Francis T.R.H.W.S., Elliott R.W. (1973). Down’s syndrome and diabetes. Psychol. Med..

[135] Bergholdt R., Eising S., Nerup J., Pociot F. (2006). Increased prevalence of Down’s syndrome in individuals with type 1 diabetes in Denmark: A nationwide population-based study. Diabetol..

[136] Burch P., Milunsky A. (1969). Early-onset diabetes mellitus in the general and down’s syndrome populations. Lancet.

[137] Carnicer J., Farré C., Varea V., Vilar P., Moreno J., Artigas J. (2001). Prevalence of coeliac disease in Down’s syndrome. Eur. J. Gastroenterol. Hepatol..

[138] Yang Q., Rasmussen S.A., Friedman J.M. (2002). Mortality associated with Down’s syndrome in the USA from 1983 to 1997: A population-based study. Lancet.

[139] Selikowitz M. (1992). Health problems and health checks in school-aged children with Down syndrome. J. Paediatr. Child Health.

[140] Cruz N.V., Mahmoud S.A., Chen H., Lowery-Nordberg M., Berlin K., Bahna S.L. (2009). Follow-up study of immune defects in patients with dysmorphic disorders. Ann. Allergy, Asthma Immunol..

[141] Klein L., Kyewski B., Allen P.M., Hogquist K.A. (2014). Positive and negative selection of the T cell repertoire: What thymocytes see (and don’t see). Nat. Rev. Immunol..

[142] Anderson M.S., Venanzi E.S., Klein L., Chen Z., Berzins S.P., Turley S.J., Von Boehmer H., Bronson R., Dierich A., Benoist C. (2002). Projection of an Immunological Self Shadow Within the Thymus by the Aire Protein. Science.

[143] LaRocca L.M., Lauriola L., Ranelletti F.O., Piantelli M., Maggiano N., Ricci R., Capelli A. (2005). Morphological and immunohistochemical study of Down syndrome thymus. Am. J. Med Genet..

[144] Lima F.A., Moreira-Filho C.A., Ramos P.L., Brentani H., Lima L.D.A., Arrais M., Bento-De-Souza L.C., Bento-De-Souza L., Duarte M.I., Coutinho A. (2011). Decreased AIRE Expression and Global Thymic Hypofunction in Down Syndrome. J. Immunol..

[145] Skogberg G., Lundberg V., Lindgren S., Gudmundsdottir J., Sandström K., Kämpe O., Annerén G., Gustafsson J., Sunnegårdh J., Van Der Post S. (2014). Altered Expression of Autoimmune Regulator in Infant Down Syndrome Thymus, a Possible Contributor to an Autoimmune Phenotype. J. Immunol..

[146] Noble J.A., Valdes A.M., Varney M.D., Carlson J.A., Moonsamy P., Fear A.L., Lane J.A., Lavant E., Rappner R., Louey A. (2010). HLA Class I and Genetic Susceptibility to Type 1 Diabetes: Results From the Type 1 Diabetes Genetics Consortium. Diabetes.

[147] Aitken R.J., Mehers K.L., Williams A.J., Brown J., Bingley P.J., Holl R.W., Rohrer T.R., Schober E., Abdul-Rasoul M.M., Shield J.P. (2012). Early-Onset, Coexisting Autoimmunity and Decreased HLA-Mediated Susceptibility Are the Characteristics of Diabetes in Down Syndrome. Diabetes Care.

[148] Miklossy J., Qing H., Radenovic A., Kis A., Vileno B., Làszló F., Miller L., Martins R.N., Waeber G., Mooser V. (2010). Beta amyloid and hyperphosphorylated tau deposits in the pancreas in type 2 diabetes. Neurobiol. Aging.

[149] Kawarabayashi T., Shoji M., Satot M., Sasaki A., Ho L., Eckman C.B., Prada C.-M., Younkin S.G., Kobayashi T., Tada N. (1996). Accumulation of β-Amyloid fibrils in pancreas of transgenic mice. Neurobiol. Aging.

[150] Matsuoka Y., Picciano M., Malester B., Lafrancois J., Zehr C., Daeschner J.M., Olschowka J.A., Fonseca M.I., O’Banion M.K., Tenner A.J. (2001). Inflammatory Responses to Amyloidosis in a Transgenic Mouse Model of Alzheimer’s Disease. Am. J. Pathol..

[151] Alexianu M.E., Kozovska M., Appel S.H. (2001). Immune reactivity in a mouse model of familial ALS correlates with disease progression. Neurol..

[152] Murphy M., Insoft R.M., Pike-Nobile L., Epstein L.B. (1995). A hypothesis to explain the immune defects in Down syndrome. Prog. Clin. Boil. Res..

[153] Feske S., Wulff H., Skolnik E.Y. (2015). Ion channels in innate and adaptive immunity. Annu. Rev. Immunol..

[154] Magge S.N., Zemel B.S., Pipan M.E., Gidding S.S., Kelly A. (2019). Cardiometabolic Risk and Body Composition in Youth With Down Syndrome. Pediatrics.

[155] Broers C.J.M., Gemke R.J.B.J., Weijerman M.E., Van Der Sluijs K.F., Van Furth A.M. (2012). Increased Pro-inflammatory Cytokine Production in Down syndrome Children Upon Stimulation with Live Influenza A Virus. J. Clin. Immunol..

[156] Rodrigues R., Debom G., Soares F., Machado C., Pureza J., Peres W., Garcias G.D.L., Duarte M.F., Schetinger M.R.C., Stefanello F. (2014). Alterations of ectonucleotidases and acetylcholinesterase activities in lymphocytes of Down syndrome subjects: Relation with inflammatory parameters. Clin. Chim. Acta.

[157] Cetiner S., Demirhan O., Inal T.C., Tastemir D., Sertdemir Y. (2010). Analysis of peripheral blood T-cell subsets, natural killer cells and serum levels of cytokines in children with Down syndrome. Int. J. Immunogenetics.

[158] Marrano N., Biondi G., Cignarelli A., Perrini S., Laviola L., Giorgino F., Natalicchio A. (2020). Fonctional loss of pancreatic islets in type 2 diabetes: How can we halt it?. Metabolism.

[159] Sorci G., Agneletti A.L., Donato R. (2000). Effects of S100A1 and S100B on microtubule stability. An in vitro study using triton-cytoskeletons from astrocyte and myoblast cell lines. Neuroscience.

[160] Gentil B.J., Delphin C., Mbele G.O., Deloulme J.C., Ferro M., Garin J., Baudier J. (2001). The Giant Protein AHNAK Is a Specific Target for the Calcium- and Zinc-binding S100B Protein. J. Biol. Chem..

[161] Arcuri C., Bianchi R., Brozzi F., Donato R. (2005). S100B Increases Proliferation in PC12 Neuronal Cells and Reduces Their Responsiveness to Nerve Growth Factor via Akt Activation. J. Biol. Chem..

[162] Tubaro C., Arcuri C., Giambanco I., Donato R. (2010). S100B protein in myoblasts modulates myogenic differentiation via NF-ÎºB-dependent inhibition of MyoD expression. J. Cell. Physiol..

[163] Mohammadzadeh F., Tsoporis J.N., Izhar S., Desjardins J.-F., Parker T.G. (2018). Deficiency of S100B confers resistance to experimental diabetes in mice. Exp. Cell Res..

[164] Peiris H., Raghupathi R., Jessup C.F., Zanin M.P., Mohanasundaram D., MacKenzie K.D., Chataway T., Clarke J.N., Brealey J., Coates P.T. (2012). Increased Expression of the Glucose-Responsive Gene, RCAN1, Causes Hypoinsulinemia, β-Cell Dysfunction, and Diabetes. Endocrinology.

[165] Helguera P., Seiglie J., Rodriguez J., Hanna M., Helguera G., Busciglio J. (2013). Adaptive Downregulation of Mitochondrial Function in Down Syndrome. Cell Metab..

[166] Shen W., Taylor B., Jin Q., Nguyen-Tran V., Meeusen S., Zhang Y.-Q., Kamireddy A., Swafford A., Powers A.F., Walker J. (2015). Inhibition of DYRK1A and GSK3B induces human β-cell proliferation. Nat. Commun..

[167] Dirice E., Walpita D., Vetere A., Meier B.C., Kahraman S., Hu J., Dančík V., Burns S.M., Gilbert T.J., Olson D.E. (2016). Inhibition of DYRK1A Stimulates Human β-Cell Proliferation. Diabetes.

[168] Wang P., Karakose E., Liu H., Swartz E., Ackeifi C., Zlatanic V., Wilson J., González B.J., Bender A., Takane K.K. (2019). Combined Inhibition of DYRK1A, SMAD, and Trithorax Pathways Synergizes to Induce Robust Replication in Adult Human Beta Cells. Cell Metab..

[169] Meier J.J., Butler A.E., Saisho Y., Monchamp T., Galasso R., Bhushan A., Rizza R.A., Butler P.C. (2008). Cell Replication Is the Primary Mechanism Subserving the Postnatal Expansion of -Cell Mass in Humans. Diabetes.

[170] Meier J.J. (2009). Linking the Genetics of Type 2 Diabetes With Low Birth Weight: A Role for Prenatal Islet Maldevelopment?. Diabetes.

[171] Song W.-J., Song E.-A.C., Jung M.-S., Choi S.-H., Baik H.-H., Jin B.K., Kim J.H., Chung S.-H. (2015). Phosphorylation and Inactivation of Glycogen Synthase Kinase 3β (GSK3β) by Dual-specificity Tyrosine Phosphorylation-regulated Kinase 1A (Dyrk1A). J. Biol. Chem..

[172] Shirakawa J., Kulkarni R.N. (2016). Novel factors modulating human β-cell proliferation. Diabetes, Obes. Metab..

[173] Butler A.E., Sacks W., Rizza R.A., Butler P.C. (2017). Down Syndrome-Associated Diabetes Is Not Due To a Congenital Deficiency in β Cells. J. Endocr. Soc..

[174] Peiris H., Duffield M.D., Fadista J., Jessup C.F., Kashmir V., Genders A.J., McGee S.L., Martin A.M., Saiedi M., Morton N. (2016). A Syntenic Cross Species Aneuploidy Genetic Screen Links RCAN1 Expression to β-Cell Mitochondrial Dysfunction in Type 2 Diabetes. PLoS Genet..

[175] Ptomey L.T., Walpitage D.L., Mohseni M., Gillette M.L.D., Davis A.M., Forseth B., Dean E.E., Waitman L.R. (2020). Weight status and associated comorbidities in children and adults with Down syndrome, autism spectrum disorder and intellectual and developmental disabilities. J. Intellect. Disabil. Res..

[176] Feki A., Hibaoui Y. (2018). DYRK1A Protein, A Promising Therapeutic Target to Improve Cognitive Deficits in Down Syndrome. Brain Sci..

